# S158. REWARD ALTERATIONS IN ANTIPSYCHOTIC NAïVE FIRST-EPISODE-PSYCHOSIS PATIENTS BEFORE AND AFTER TREATMENT WITH A PARTIAL DOPAMINE AGONIST

**DOI:** 10.1093/schbul/sby018.945

**Published:** 2018-04-01

**Authors:** Karen Tangmose, Mette Odegård Nielsen, Anne Sigvard, Kasper Jessen, Kirsten Bojesen, Marie Bjerregaard, Egill Rostrup, Birte Glenthoj

**Affiliations:** 1 Center for Neuropsychiatric Schizophrenia Research; 2 Center for Neuropsychiatric Schizophrenia Research, CNSR, and Center for Clinical Intervention and Neuropsychiatric Schizophrenia Research, CINS, Mental Health Center Glostrup, University of Copenhagen

## Abstract

**Background:**

Alterations of the brain reward system is a common finding in patients with psychoses and it may be affected by antipsychotic medication. There are however only few longitudinal studies on medication effect and the effect of a partial dopamine agonist have not previously been examined in patients. The aim of the present study is to explore reward abnormalities in first episode psychotic patients and matched heathy controls (HC) before and after treatment with a partial dopamine agonist (aripiprazole), and relate the findings to dopamine synthesis capacity (F-DOPA-PET), glutamate and GABA levels in the brain (MRS at 3T) and treatment outcome. Here we present preliminary baseline and follow up analyses on functional magnetic resonance imaging (fMRI) only.

**Methods:**

The project is a part of a multimodal prospective cohort study. Reward related brain activity was examined with fMRI using a variant of the Monetary Incentive Delay Task before and after 6 weeks, where patients were treated with individual doses of aripiprazole. Psychopathology was measured with the Positive and Negative Syndrome Scale (PANSS). Whole brain voxel-wise group comparison was performed at baseline and follow up using two sample t-test with a corrected cluster significant threshold of P=0.05. Likewise, the effect of time and group time interaction was analyzed voxel-wise.

**Results:**

Inclusion is ongoing and data have been analyzed for 19 patients, age 22.9(4.6), 9 males (47%) and 24 HC, age 22.1(2.7), 11 males (46%). Mean medication dose was 11.7 (6) mg aripiprazole at follow up.

Psychopathology: At baseline patients were moderately ill with a mean PANSS total score of 69 (14). Paired t-test showed a significant reduction over time for PANSS total score to 57 (12) (P<0.001), with significant improvements in PANSS positive, PANSS negative and PANSS general scores (all p<0.05).

fMRI: There were no group differences at baseline.

At follow up, patients had an increased signal in medial frontal cortex and Anterior Cingulate Cortex (ACC) compared to HC during anticipation of monetary gain. During outcome evaluation, patients likewise had an increased signal in right striatum and paracingulate gyrus in the win contrast, increased signal in left ventral part of striatum and ACC in the lose contrast, and increased signal in right striatum and ACC in the miss contrast compared to HC. There was only a significant effect of time in patients in the anticipation to win contrast and no significant group time interaction.

**Discussion:**

The data represent work in progress and should be taken with precaution. The group-differences at follow up which were not found at baseline may suggest that treatment with a partial dopamine agonist lead to alterations of reward processing in patients. This is further supported by the effect of time in patients in the anticipation to win contrast. The data collection is still ongoing, and we expect to increase the size of the cohort and plan to relate the findings to measures of dopamine, GABA, glutamate and psychopathology.

